# 1273. Use of an algorithm-based electronic application in the management of penicillin allergies

**DOI:** 10.1093/ofid/ofad500.1113

**Published:** 2023-11-27

**Authors:** Nimal Vijayaraghavan, Ohide Otome

**Affiliations:** Rockingham General Hospital, Perth, Western Australia, Australia; SJOG Midland Private and Public Hospital, Perth, Western Australia, Australia

## Abstract

**Background:**

Penicillin allergy "de-labelling" has shown to improve antibiotic stewardship. Knowledge gaps of antibiotic allergies amongst healthcare workers are well known. We attempt to evaluate the use of an algorithm-based electronic application as a point of care (POC) decision making tool in penicillin allergy management.

**Methods:**

A 4 week prospective, randomized case-control study was conducted on healthcare workers in a tertiary care centre. Local general practitioners were included. Participants were randomized to the control and experimental arms. Both were required to complete a set of 8 case-based questions specific to penicillin reactions. The former was given access to an electronic application, while the latter were permitted to use alternative educational tools. Time of completion in one sitting and total number of correct answers of both groups were obtained and compared. The experimental arm's overall impressions of the electronic algorithm were defined by completion of an optional satisfaction survey. The assessment tools were created using REDcap software.

Algorithm-based electronic application

**Figure:**
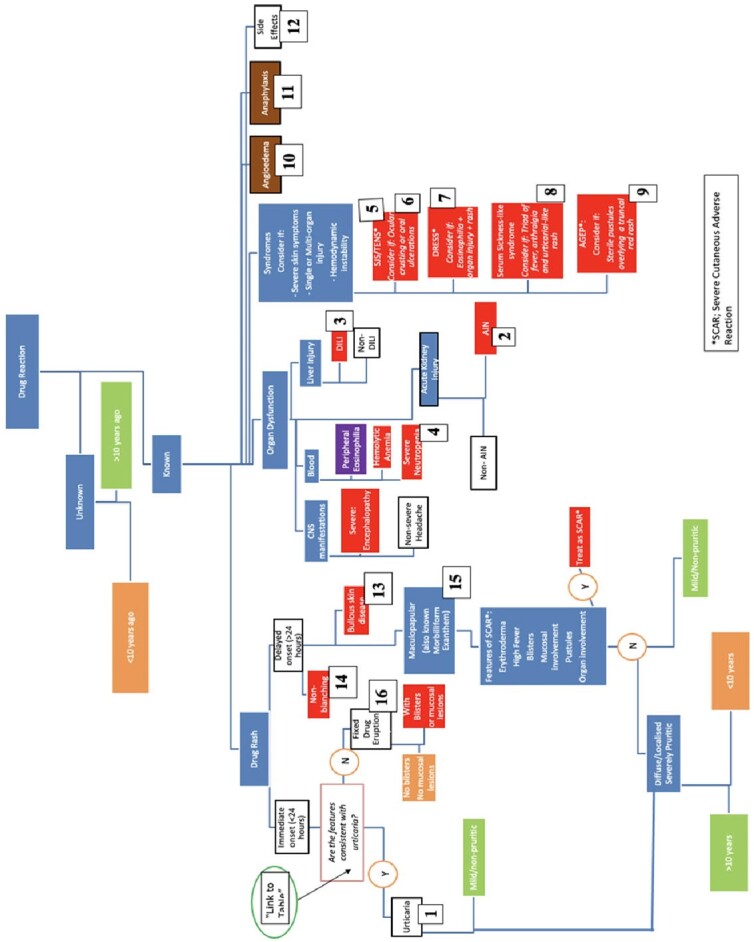

The algorithm-based electronic application was made available to the experimental arm to aid in completion of the questionnaire.

Case-based questionnaire

**Figure:**
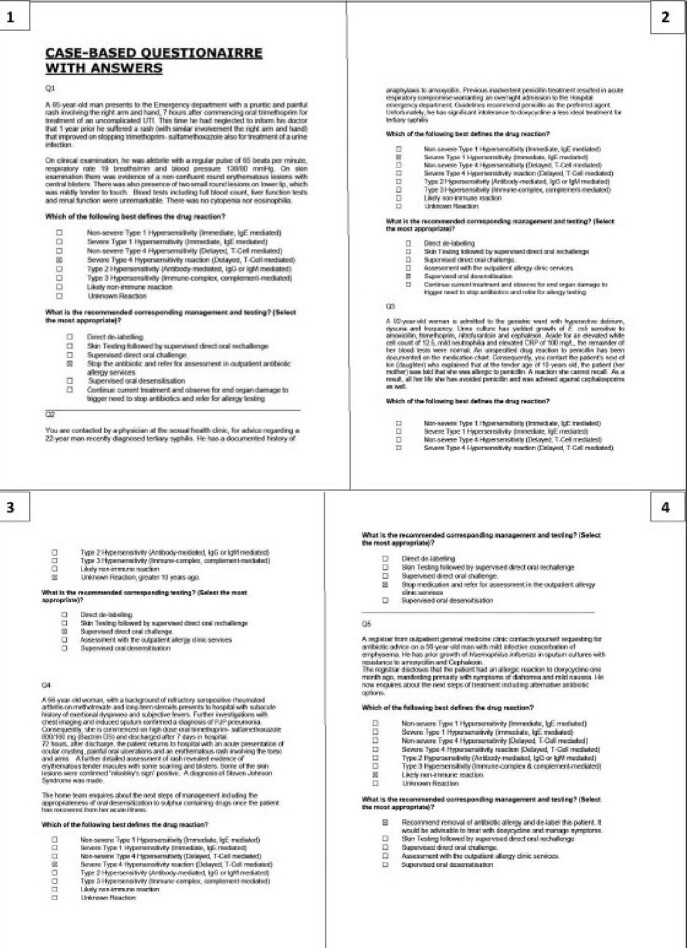

All candidates in the study were required to complete an assessment composed of 8 scenario-based questions (16 questions total) to evaluate the clinical understanding of antibiotic allergies.

**Results:**

Only 68 out of 347 eligible healthcare workers responded to the invite to the study and questionnaire. From the selected group, 31 and 37 participants were randomized to the control and experimental arms respectively. Majority of participants were hospital-based practitioners (85%). A higher completion rate of the questionnaire was observed in the experimental group (73% vs 65%). The experimental arm completed 86% of the total allocated questions (516 out of 592) compared to 78% (386 out of 496) in the control (p< 0.001). Further observation revealed that the experimental group achieved correct answers in 69% (351) of the total attempted questions in contrast to 59% (228) correct answers in the control (p< 0.01). No statistically significant difference in mean times of completion were observed between the groups (p = 0.25). With regards to the survey, 96% found the electronic application useful, 84% would strongly recommend it to their peers, 88% would like to have access to it to support them in their POC decisions.

**Conclusion:**

In this study, we demonstrate that an electronic POC allergy delabelling application may have the potential to support clinicians in the management of patients reporting penicillin allergy.

**Disclosures:**

**All Authors**: No reported disclosures

